# Supplementary material for: Swaying slower reduces the destabilizing effects of a compliant surface on voluntary sway dynamics
Source: PLoS One. 2019 Dec 11;14(12):e0226263. doi: 10.1371/journal.pone.0226263 (PMC6905565; doi:10.1371/journal.pone.0226263)
Supplement: Attachment — Submitted filename: Review of PONE.pdf [file pone.0226263.s001.pdf]

Review of PONE-D-19-17729, "Swaying slower reduces the destabilizing effects of a compliant surface on voluntary sway dynamics, for *PLOS ONE*. August 2019.

The authors focus on muscle activation, but do not give a motivation for this choice. The choice is odd given the inclusion of data on movement dynamics. In revising, it is essential that they address a fundamental problem that arises with data on muscle activation: There is not a 1:1 relation between muscle activity and movement kinematics. A given pattern of muscle activation can yield different movement outcomes, and a given movement outcome can be achieved using different muscle activation patterns. This well-documented fact was central to Bernstein's critique of classical approaches to the study of motor control. Outside the clinical literature, it now is widely accepted that patterns of muscle activation are not of central importance in motor control. That is, it is widely accepted that what the nervous system actually controls patterns of coordination (e.g., ankle-hip relative phase), and not patterns of muscle activation. Given this very large literature, why is it appropriate to focus on muscle activation?

The focus on muscle activation is strange, also, because the authors collected data on postural kinematics, that is, the position of the COP. Thus, the purpose stated in lines 79-80 appears to not accurately reflect the study that was conducted. Indeed, most of the data reported are analyses of COP kinematics, and not EMG. Critically, the authors offer no analysis of how the COP kinematics and the EMG data might be related to each other. Again, the central question concerns the lack of an explicit motivation to study EMG data, given that general literature and the effects (in the current study) found in analyses of COP kinematics.

The authors assert, "voluntary CoP shifting is necessary for everyday activities". This assertion is problematic because the term, *voluntary*, is not defined. Voluntary might mean something such as "self-initiated". Or, voluntary might mean something relating to conscious awareness or intention. There is no question that everyday activities include a great deal of self-initiated postural adjustments. It is much more controversial (and, almost certainly wrong) to suggest that these activities include conscious awareness or intent. It is essential that the authors be very clear about this distinction, especially due to the fact that, in the present study, participants performed an explicit task, in which postural adjustments came from conscious intention. In this critical sense, the study appears to deviate qualitatively from "everyday activities".

With respect to margins of stability, it would be helpful if the authors could contrast their view (which is based upon McCollum & Leen), with the growing body of research documenting the perceptual-motor reality of postural time-to-contact (e.g., Haddad et al., 2006; Slobounov et al., 1997, 1998; van Wegen et al., 2002), which is based upon Riccio & Stoffregen (1988; Riccio, 1993). Note, in particular, that postural time-to-contact cannot be defined in terms of muscle activation.

Haddad, J. M., Gagnon, J. L., Hasson, C. J., Emmerik, R. E. A. V., & Hamill, J. (2006). Evaluation of time-to-contact measures for assessing postural stability. *Journal of Applied Biomechanics*, *22*, 155-161.

Riccio, G. E. (1993). Information in movement variability about the qualitative dynamics of posture and orientation. In K. M. Newell & D. M. Corcos (Eds.), *Variability and motor control* (pp. 317-357). Champaign IL: Human Kinetics Publishers.

Riccio, G. E., & Stoffregen, T. A. (1988). Affordances as constraints on the control of stance. *Human Movement Science, 7,* 265-300.

Slobounov, S. M., Moss, S. A., Slovounova, E. S., & Newell, K. M. (1998). Aging and time to instability in posture. *Journals of Gerontology: Biological Sciences*, *53A*, B71-B78.

Slobounov, S. M., Slobounova, E. S., & Newell, K. M. (1997). Virtual time-to-collision and human postural control. *Journal of Motor Behavior*, *29*, 263–281.

Van Wegen, E. E., van Emmerik, R. E. A., & Riccio, G. E. (2002). Postural orientation: Age-related changes in variability and time-to-boundary. *Human Movement Science*, *21*, 61-84.
