# Supplementary material for: Swaying slower reduces the destabilizing effects of a compliant surface on voluntary sway dynamics
Source: PLoS One. 2019 Dec 11;14(12):e0226263. doi: 10.1371/journal.pone.0226263 (PMC6905565; doi:10.1371/journal.pone.0226263)
Supplement: Attachment — Submitted filename: Response1.docx [file pone.0226263.s002.docx]

**Response to reviewers**

We would like to thank the reviewers for their thoughtful comments and the raised concerns which motivated us to improve the quality of this work.

**Reviewer #1**

*The study is interesting; however, some minor concerns might need to be clarified.*

1. *The manuscript mention that seventeen volunteers performed a 2-min voluntary sway task (page 2), and each trail lasted 2 minutes with 2-3 minutes interval in between…no longer than one hour (page 7). How many trails for each volunteer in the study? Is there any pre-trial for subjects to familiar the device?*

The preparation for electrode and marker placement lasted approximately 15 minutes (it also included the placement of an ultrasound head). Prior to measurement a familiarization session was performed (~3 minutes). Each participant performed one 2-minute trial on each of the four conditions with 2-3 minutes interval in between (16-20 minutes). Two more conditions (without CoP feedback and with a target other than sinusoidal) were included in the current experiment which added another 8-10 minutes. Data from these conditions and the ultrasound measurements are not presented here, because they focus on different research questions. In total, this makes 42-54 minutes (15 + 3 + 2X2X6 or 15 + 3 + 2X3X6). To clarify these issues, we specified in the manuscript the following: “The session, including the subject preparation (15 minutes), lasted no longer than one hour, considering the set-up for ultrasound recordings and two additional conditions (data not presented here).” (L155-157).

2. *What is the function of the 2 dots (yellow and red) mentioned in the page 6? By the way, what is the purpose of the experimental design showed in figure 1 (A)?*

This experimental paradigm has been previously used (4). Its purpose is to create a visually-guided weight-shifting task for controlling voluntary the COP in the anterior-posterior direction. Both dots were restricted to vertical movement, in the middle of the screen. Each of them served a different purpose. The yellow dot displayed the real-time position of CoP at the anterior/posterior direction. The red dot was the target that the participant should overlap with the yellow dot, by moving his/her CoP in the anterior/posterior direction (see also L121-127). To clarify the purpose of this experimental design we added the following sentence in the manuscript: “The following experimental paradigm was used to create a visually guided weight-shifting task for controlling voluntary the CoP in the anterior-posterior direction.” (L120-121).

3. *What is target signal and how to capture and estimate?*

The target (red dot) was a time-series sinusoidal signal, generated by a sine wave function with a fixed time period for each condition (either 4 or 2 s, to create an oscillation at 0.25 or 0.125 Hz, respectively). The sample frequency was 50 Hz, i.e. one data point every 20 ms, which resulted in 8000 and 4000 samples per cycle for the 0.25 and 0.125 Hz conditions, respectively. This information has been added in the methods: “The target (red dot) was a time-series sinusoidal signal, generated by a sine wave with a fixed time period for each condition (either 4 or 2 s, to create an oscillation at 0.25 or 0.125 Hz, respectively), with a sample frequency of 50 Hz (i.e. one data point every 20 ms, which resulted in 8000 and 4000 samples per cycle for the 0.25 and 0.125 Hz conditions, respectively).” (L131-135).

The amplitude of the sinusoidal signal (range between minimum and maximum) was set at 60% of foot length. The midpoint (zero value) of the signal was set at the mean CoP_AP_ position when the participant reached his/her most anterior and posterior position without moving the feet from the ground. Each data sample of this signal was fed to the vertical position of the red dot on the monitor at 50 frames per second. To make it clearer to the reader, the above information that was missing has been added in the text: “The amplitude of the target movement (red dot) was set at 60% of the foot length, with zero representing the midpoint of the CoP_AP_ range when the participant was leaning as much as possible anteriorly and posteriorly without moving the feet from the ground (Fig 1B).” (L137-140).

4. *The values showed in table 1 by the joint kinematics (Ankle, knee, hip, spine and haed). How these values be measured and estimated? And what are the meanings for these joint and head?*

The norm values of for the x, y and z space dimensions were calculated as $N_{i}=\sqrt{X_{i}^{2}+Y_{i}^{2}+Z_{i}^{2}}$, where N_i_ is the norm value of the i^th^ datapoint with coordinates X, Y, and Z on the x, y and z axis, respectively. The maximum Lyapunov exponent (MLE) for the ankle, knee, hip, spine and head were calculated using the norm coordinates of markers placed on the lower limbs, trunk and head. More specifically, for the lower limbs the analyzed coordinates were defined as the midpoint between the markers attached to the two sides of the body. For the ankle, markers were placed on the lateral malleoli, for the knee on the lateral epicondyles, and for the hip on the greater trochanter. For the spine, the norm coordinates of a marker on the 7^th^ cervical vertebra were analyzed, and for the head the norm coordinates of 4 markers placed around the head (two on the forehead and two on the back of the head while wearing a headband). In the revised manuscript we explained the procedure clearer by adding the following text “The 3-dimensional coordinate data of the ankles (lateral malleoli), knees (lateral epicondyles), hips (greater trochanters), spine (7^th^ cervical vertebra) and head (4 markers around the head placed on a headband) were acquired at 250 Hz and have been filtered in the same manner. All kinematic data were subsequently down-sampled to 15,000, and we calculated the midpoint between the two sides of the body and the midpoint of the 4 head markers. The 3-dimensional coordinates of these virtual markers in addition to the marker at spine were used for the MLE calculation.” (L193-199).

Furthermore, we apologize for mistakenly stating that the CoP_AP_ was analyzed for the MLE; we calculated the norm value of the anterior-posterior and mediolateral axes (see corrections substituting CoP_AP_ with CoP in L189, L190, L245, L250/Table 1, L328, and L356).

5. *In figure 2, what is the unit in vertical axis?*

The values of CoP_AP_ displacement on the vertical axis are expressed as percent of foot length. This is corrected and now shown in Figure 2.

6. *In figure 3, the pink circle and blue triangle mean the average values?*

All large circles and triangles represent the group means. Individual values of each participant are shown with small symbols (circles and triangles). Some modifications/corrections in the legend may now help in understanding: “Pink circles connected with continuous lines and blue triangles connected with dashed lines represent the condition of sway on rigid ground and foam, respectively. Small and large symbols (circles and triangles) represent data of each individual and group means, respectively. Vertical lines designate one standard deviation and asterisks express the presence of significant main effect (p<0.05).” (L271-274).

7. *Figure 5 show the typical example of EMG signal by the figure legend (page 27), however, it needs more explanations to realize the four figures mean.*

Figure 5 displays a typical example of EMG data from one participant, in all four examined conditions. The condition depicted on each graph is written on the left side of each graph, namely from top to bottom, swaying on rigid surface with natural sway frequency, swaying on foam with natural frequency, swaying on rigid surface with slow frequency, and swaying on foam with slow frequency. To clarify this, we added in the figure caption “Each of the four graphs represents one condition as described on the left side of the graph.” (L309-310).

8. *Can the authors quantify a clear threshold or standard to define a sway frequency range for rehabilitation programs in the conclusion by the results?*

This is an interesting question but very hard to answer with the current set up. We examined only two frequencies, and this gives us no confidence to define a threshold for a sway frequency range in rehabilitation programs. In the literature sway frequencies ranging from 0.2 to 1 Hz have been reported, with different effects on the limits of stability, visuo-motor coupling strength and postural strategies (2,7–9). Several studies from our lab and ample of pilot testing suggests that a natural and inherently stable voluntary sway frequency is between 0.25 and 0.30 Hz. However, this is subject of individual variations stemming from anthropometric differences and variations in rhythm perception. Thus, a reasonable suggestion could be scaling sway frequency based on the individual internal rhythm of self-paced sway depending on the movement parameter that is aimed to be improved, but certainly, this question could be a subject for future studies.

**Reviewer #2**

1. *The authors focus on muscle activation, but do not give a motivation for this choice. The choice is odd given the inclusion of data on movement dynamics. In revising, it is essential that they address a fundamental problem that arises with data on muscle activation: There is not a 1:1 relation between muscle activity and movement kinematics. A given pattern of muscle activation can yield different movement outcomes, and a given movement outcome can be achieved using different muscle activation patterns. This well-documented fact was central to Bernstein’s critique of classical approaches to the study of motor control. Outside the clinical literature, it now is widely accepted that patterns of muscle activation are not of central importance in motor control. That is, it is widely accepted that what the nervous system actually controls patterns of coordination (e.g., ankle-hip relative phase), and not patterns of muscle activation. Given this very large literature, why is it appropriate to focus on muscle activation?*

We would like to thank the reviewer for this comment and critique to our approach. The decision to measure and present EMG data in addition to the movement dynamics was a key-element of the study. However, it was *not* our intent to examine what the nervous system controls by means of EMG measures. Despite its numerous methodological limitations, EMG can give a rough representation of the activation patterns during movement and help us understand the underlying mechanisms of movement regulation. We agree that this activation - movement relationship is not 1:1, and that it is not possible do depict movement with EMG data. EMG can assess muscle activation, however the resulted muscle force that is responsible for the kinematic changes depends on other mechanisms as well, such as the force-length and force-velocity relationship. Independent of which parameters determine the final movement outcome, the basic activation patterns of the muscles for a target movement are initiated from the central nervous system and are linked to the target movement. This study attempts to improve our understanding of neuromuscular mechanisms in the presence of instability (i.e. postural sway on an unstable surface) by describing the basic activation patterns of 13 superficial muscles during a relatively simple cyclical task, constrained to an oscillation around the ankle joint. By averaging multiple cycles of EMG and CoP patterns, we reduce the inherent variability between cycles and although we lose the detailed information about each sway cycle, we can have a broader picture about the general behavior of the system on stable and unstable surface, with two different swaying frequencies. We are confident that the EMG activation patterns underlying such a task will help us to understand neuromuscular mechanisms and strategies that control voluntary sway.

2. *The focus on muscle activation is strange, also, because the authors collected data on postural kinematics, that is, the position of the COP. Thus, the purpose stated in lines 79-80 appears to not accurately reflect the study that was conducted. Indeed, most of the data reported are analyses of COP kinematics, and not EMG. Critically, the authors offer no analysis of how the COP kinematics and the EMG data might be related to each other. Again, the central question concerns the lack of an explicit motivation to study EMG data, given that general literature and the effects (in the current study) found in analyses of COP kinematics.*

In our study using kinematic data and non-linear time series analysis (i.e. MLE) we quantify the local stability of the human system during the investigated task and chosen conditions. By adding the EMG data, we intended to describe the consequences of our experimental manipulations in the neuromuscular organization and movement effectivity. More specifically, although training on unstable surfaces seems to have beneficial effects on balance in older individuals (3,5) and patients (6,10), we still do not know what the effects on the stability of the system are, how it affects movement, and how the muscles respond to this perturbed state. For a better description of the purpose of the study we modified the respective sentence: “Considering the above, the main purpose of this study is to manipulate the surface stability (i.e. standing on firm ground or on foam) and the execution speed (i.e. two different sway frequencies) examine the effects on manipulating surface stability and execution speed during voluntary, visually guided voluntary postural sway.” (L82-85)

Regarding the lack of any analysis supporting a connection of the COP kinematics with the EMG data, as we mentioned above (Reviewer 2, point 1), muscle activation cannot accurately predict force (which produces movement), due to the effects of the force-length and force-velocity relationship. However, when the contraction is isometric the force/EMG relationship is more predictable (1). Having partially analyzed data from ultrasound recordings from this experimental setup (not presented here) we are quite confident that the muscles contract almost in an isometric fashion during this task. Keeping this in mind, we calculated the correlation coefficients between the CoP_AP_ and the EMG of each muscle. The following table shows the correlation coefficients for the whole cycle and for the first and second half cycle separately (since most of the muscles demonstrated no diphasic activation). Values over 0.9 and below -0.9 are shown in boldface and the last line shows as reference the correlation coefficient of COP_AP_ with the target.

|  | **Full cycle** | | | |
| --- | --- | --- | --- | --- |
|  | **Slow & Rigid** | **Slow & Foam** | **Natural & Rigid** | **Natural & Foam** |
| GlMed | 0.758 | -0.091 | 0.720 | -0.267 |
| GlMax | 0.414 | -0.300 | 0.388 | -0.241 |
| TFsL | 0.335 | -0.512 | -0.081 | -0.449 |
| RF | **-0.921** | **-0.910** | -0.891 | -0.859 |
| VM | **-0.912** | **-0.916** | -0.897 | -0.858 |
| VL | **-0.911** | **-0.924** | -0.897 | -0.860 |
| Sem | **0.969** | **0.937** | **0.942** | 0.868 |
| BF | 0.737 | -0.066 | 0.182 | 0.015 |
| TA | -0.856 | -0.860 | -0.893 | -0.837 |
| PL | **0.938** | 0.743 | **0.925** | 0.610 |
| GM | **0.968** | **0.956** | **0.930** | 0.884 |
| GL | **0.964** | **0.934** | **0.964** | 0.834 |
| Sol | **0.989** | **0.968** | **0.954** | 0.881 |
| Target | **0.995** | **0.989** | **0.989** | **0.991** |

|  | **First half cycle** | | | | **Second half cycle** | | | |
| --- | --- | --- | --- | --- | --- | --- | --- | --- |
|  | **Slow & Rigid** | **Slow & Foam** | **Natural & Rigid** | **Natural & Foam** | **Slow & Rigid** | **Slow & Foam** | **Natural & Rigid** | **Natural & Foam** |
| GlMed | 0.854 | 0.833 | 0.680 | 0.643 | -0.289 | -0.822 | 0.012 | -0.819 |
| GlMax | 0.471 | 0.585 | 0.664 | -0.244 | -0.291 | -0.501 | -0.152 | -0.387 |
| TFsL | 0.777 | 0.741 | 0.403 | 0.832 | -0.773 | -0.801 | -0.406 | -0.736 |
| RF | -0.710 | -0.584 | -0.767 | 0.121 | **-0.959** | **-0.942** | -0.770 | -0.779 |
| VM | -0.824 | -0.795 | -0.716 | 0.128 | **-0.932** | **-0.929** | -0.702 | -0.793 |
| VL | -0.795 | **-0.922** | -0.649 | 0.127 | **-0.949** | **-0.938** | -0.759 | -0.802 |
| Sem | **0.935** | 0.856 | 0.858 | 0.629 | 0.863 | 0.446 | 0.656 | 0.268 |
| BF | 0.805 | 0.789 | 0.772 | **0.918** | -0.477 | **-0.913** | -0.495 | -0.724 |
| TA | 0.374 | 0.119 | -0.538 | 0.219 | **-0.968** | -0.877 | -0.887 | -0.771 |
| PL | **0.958** | **0.917** | **0.931** | 0.757 | 0.439 | -0.606 | 0.302 | -0.474 |
| GM | **0.912** | 0.817 | 0.749 | 0.489 | 0.817 | 0.731 | 0.640 | 0.559 |
| GL | **0.952** | 0.790 | 0.897 | 0.424 | 0.891 | 0.704 | 0.807 | 0.401 |
| Sol | **0.969** | 0.883 | 0.787 | 0.541 | **0.931** | 0.818 | 0.802 | 0.537 |
| Target | **0.976** | **0.949** | **0.969** | **0.987** | **0.982** | **0.986** | **0.921** | **0.932** |

However, despite this analysis which reveals a relationship between these EMG and COP_AP_, especially during the slow sway, it is not the scope of this work to focus or even support the notion that the EMG data have a direct relationship with the movement COP_AP_. Therefore, we would prefer not to include this analysis in the manuscript.

3. *The authors assert, “voluntary CoP shifting is necessary for everyday activities”. This assertion is problematic because the term, voluntary, is not defined. Voluntary might mean something such as “self-initiated”. Or, voluntary might mean something relating to conscious awareness or intention. There is no question that everyday activities include a great deal of self-initiated postural adjustments. It is much more controversial (and, almost certainly wrong) to suggest that these activities include conscious awareness or intent. It is essential that the authors be very clear about this distinction, especially due to the fact that, in the present study, participants performed an explicit task, in which postural adjustments came from conscious intention. In this critical sense, the study appears to deviate qualitatively from “everyday activities”.*

Thank you for this comment. The reason for using the term “voluntary” was to discriminate sway from the spontaneous sway we observe during natural quiet standing, but this seems to be misleading. We agree with the reviewer that the notion “voluntary” is not well defined and might implicate different interpretations. We should draw a line between intended voluntary activities that require CoP manipulation to execute gait initiation and to stand up from a chair and the corrective but still voluntary activation during perturbations, i.e. unpredicted situations. In the latter case, “self-initiated” movements may also occur, but these belong to the reflexes. In the present study, we do use an explicit task (visually guided anterior-posterior postural sway), but we also combine this task with a perturbed state, i.e. standing on foam. When standing on foam, there is a conscious intention to maintain balance. We do not imply that this is an “everyday activity”, but this is an experimental paradigm that can show how the system adapts to continuous and partly unpredictable balance perturbations. To clarify this issue, we modified the relevant text in the manuscript as follows: “Shifting body weight is necessary for everyday activities, such as gait initiation or getting up from a chair and might be crucial under externally imposed sensory constraints (e.g. when avoiding an obstacle or stepping on a slippery surface).” (L66-68).

4. *With respect to margins of stability, it would be helpful if the authors could contrast their view (which is based upon McCollum & Leen), with the growing body of research documenting the* *perceptual-motor reality of postural time-to-contact (e.g., Haddad et al., 2006; Slobounov et al., 1997, 1998; van Wegen et al., 2002), which is based upon Riccio & Stoffregen (1988; Riccio, 1993). Note, in particular, that postural time-to-contact cannot be defined in terms of muscle activation.*

We thank the reviewer for pointing us to this interesting body of evidence addressing an alternative theoretical view that we were not aware of. From a perception-action point of view, participants intuitively reduce the temporal proximity to the margin of stability when a) sway velocity increases b) base of support decreases and c) the degrees of freedom are reduced (12,13). This might explain the target undershooting in the foam condition compared to the rigid surface and the temporal delay (i.e. phase shift between the target and the CoP motion) in the natural frequency compared to the slow one.

To incorporate the suggested theoretical perspective in our results interpretation, we added the following paragraph in the discussion after reading and considering the suggested literature: “An alternative explanation of the stronger temporal coupling during slow compared to natural sway and the stronger spatial coupling when standing on rigid ground compared to the foam surface could stem from the perceptual-motor reality of postural time-to-contact [52,53]. According to this concept, the temporal proximity to the margin of stability is reduced intuitively when a) sway velocity increases b) the base of support decreases and c) the degrees of freedom are reduced [54]. Regarding the findings of the present study, it could be argued that one reason for the weaker temporal coupling (greater phase lag) in the higher than the lower sway frequency might be the greater movement velocity that induced a reduction in the margins of stability. This is also reflected by the fact that MLE was increased only distally (CoP and ankle) when the surface of support became unstable at slower sway frequencies, whereas the consequences of the foam surface on the stability of the system emerged up to the head when swaying at higher frequencies.” (L379-389).

**Response to the journal’s requirements**

*Please ensure that your manuscript meets PLOS ONE's style requirements, including those for file naming. The PLOS ONE style templates can be found at* [*http://www.journals.plos.org/plosone/s/file?id=wjVg/PLOSOne_formatting_sample_main_body.pdf*](http://www.journals.plos.org/plosone/s/file?id=wjVg/PLOSOne_formatting_sample_main_body.pdf) *and* [*http://www.journals.plos.org/plosone/s/file?id=ba62/PLOSOne_formatting_sample_title_authors_affiliations.pdf*](http://www.journals.plos.org/plosone/s/file?id=ba62/PLOSOne_formatting_sample_title_authors_affiliations.pdf)

The manuscript has been revised regarding the style requirements. Furthermore, all figures have been uploaded and proofed in PACE digital diagnostic tool and as a consequence, Figures 2 & 3 specifications have been updated.

*Please provide additional details regarding participant consent also in the ethics statement (currently you only describe consent given in the methods section). In the ethics statement in the online submission information, please ensure that you have specified (1) whether consent was informed and (2) what type you obtained (for instance, written or verbal, and if verbal, how it was documented and witnessed). If your study included minors, state whether you obtained consent from parents or guardians. If the need for consent was waived by the ethics committee, please include this information.*

The consent was written. This has been added in the revised manuscript (L102). Furthermore, we added in the online submission for that “The consent was informed in written form, and the study did not include any minors.”.

Literature

1.	Alkner BA, Tesch PA, Berg HE. Quadriceps EMG/force relationship in knee extension and leg press. Med Sci Sports Exerc 2000;32:459–63.

2.	Danion F, Duarte M, Grosjean M. Variability of reciprocal aiming movements during standing : The effect of amplitude and frequency. Gait Posture 2006;23:173–9. doi:10.1016/j.gaitpost.2005.01.005.

3.	Hamed A, Bohm S, Mersmann F, Arampatzis A. Exercises of dynamic stability under unstable conditions increase muscle strength and balance ability in the elderly. Scand J Med Sci Sports 2018;28:961–71. doi:10.1111/sms.13019.

4.	Hatzitaki V, Stergiou N, Sofianidis G, Kyvelidou A. Postural sway and gaze can track the complex motion of a visual target. PLoS One 2015;10:1–14. doi:10.1371/journal.pone.0119828.

5.	Hirase T, Inokuchi S, Matsusaka N, Okita M. Effects of a Balance Training Program Using a Foam Rubber Pad in Community-Based Older Adults. J Geriatr Phys Ther 2015;38:62–70. doi:10.1519/JPT.0000000000000023.

6.	Hung J-W, Chou C-X, Hsieh Y-W, Wu W-C, Yu M-Y, Chen P-C, et al. Randomized Comparison Trial of Balance Training by Using Exergaming and Conventional Weight-Shift Therapy in Patients With Chronic Stroke. Arch Phys Med Rehabil 2014;95:1629–37. doi:10.1016/j.apmr.2014.04.029.

7.	Lorenzo TM, Vanrenterghem J. Effects of increased anterior-posterior voluntary sway frequency on mechanical and perceived postural stability. Hum Mov Sci 2015;39:189–99. doi:10.1016/j.humov.2014.11.012.

8.	Marin L, Bardy BG, Baumberger B, Flückiger M, Stoffregen TA. Interaction between task demands and surface properties in the control of goal-oriented stance. Hum Mov Sci 1999;18:31–47.

9.	Murnaghan CD, Elston B, Mackey DC, Robinovitch SN. Modeling of postural stability borders during heel–toe rocking. Gait Posture 2009;30:161–7. doi:10.1016/J.GAITPOST.2009.03.010.

10.	Page P. Sensorimotor training: A “global” approach for balance training. J Bodyw Mov Ther 2006;10:77–84. doi:10.1016/J.JBMT.2005.04.006.

11.	Riccio GE, Stoffregen TA. Affordances as constraints on the control of stance. Hum Mov Sci 1988;7:265–300.

12.	Slobounov SM, Moss SA, Slobounova ES, Newell KM. Aging and time to instability in posture. J Gerontol A Biol Sci Med Sci 1998;53:B71-8. doi:10.1093/gerona/53a.1.b71.

13.	Slobounov SM, Slobounova ES, Newell KM. Virtual Time-to-Collision and Human Postural Control. J Mot Behav 1997;29:263–81. doi:10.1080/00222899709600841.
