# Supplementary material for: Swaying slower reduces the destabilizing effects of a compliant surface on voluntary sway dynamics
Source: PLoS One. 2019 Dec 11;14(12):e0226263. doi: 10.1371/journal.pone.0226263 (PMC6905565; doi:10.1371/journal.pone.0226263)
Supplement: Attachment — Submitted filename: Response.docx [file pone.0226263.s003.docx]

**Response to reviewers**

We would like to thank the reviewers for their comments on the revised manuscript. It is also appreciated that our efforts to improve the content of our work has been in a good extent acknowledged.

**Reviewer #1**

*The manuscript has been revised and all comments have been addressed completely by the authors' responses.*

Thank you for the positive comment regarding our revised manuscript.

**Reviewer #2**

*The authors Response to Reviews is good. Unfortunately, not much of that actually appears in the revised manuscript. The paragraph beginning Line 82 offers a prediction about EMG, but makes no other predictions, and says nothing about why it is useful to combine data on EMG with the various types of kinematic data. The impression continues to be that of a fishing expedition.*

Thank you for the encouraging remarks regarding our first revision. We amended in the introduction a sentence that made our hypothesis more specific with the other than the EMG variables: “It is expected that swaying at a lower frequency may improve coherence between the target and the CoP when standing on unstable ground and this might be accompanied with changes in the activation of the lower limb muscles.” (L85-87).
